# Ketogenic diet ameliorates cognitive impairment and neuroinflammation in a mouse model of Alzheimer’s disease

**DOI:** 10.1111/cns.13779

**Published:** 2021-12-10

**Authors:** Yunlong Xu, Chenyu Jiang, Junyan Wu, Peidong Liu, Xiaofei Deng, Yadong Zhang, Bo Peng, Yingjie Zhu

**Affiliations:** ^1^ Shenzhen Key Laboratory of Drug Addiction Shenzhen Neher Neural Plasticity Laboratory the Brain Cognition and Brain Disease Institute (BCBDI) Shenzhen Institute of Advanced Technology Chinese Academy of Sciences (CAS) Shenzhen‐Hong Kong Institute of Brain Science‐Shenzhen Fundamental Research Institutions Shenzhen China; ^2^ University of Chinese Academy of Sciences Beijing China; ^3^ The First Affiliated Hospital Sun Yat‐sen University Guangzhou China; ^4^ Medical College of Acupuncture‐Moxibustion and Rehabilitation Guangzhou University of Chinese Medicine Guangzhou China; ^5^ State Key Laboratory of Medical Neurobiology MOE Frontiers Center for Brain Science Institute for Translational Brain Research Fudan University Shanghai China; ^6^ Faculty of Life and Health Sciences Shenzhen Institute of Advanced Technology Chinese Academy of Sciences Shenzhen China; ^7^ CAS Center for Excellence in Brain Science and Intelligence Technology Chinese Academy of Sciences Shanghai China; ^8^ CAS Key Laboratory of Brain Connectome and Manipulation The Brain Cognition and Brain Disease Institute (BCBDI) Shenzhen Institute of Advanced Technology (SIAT) Chinese Academy of Sciences (CAS) Shenzhen China

**Keywords:** Alzheimer's disease, cognitive impairment, ketogenic diet, microglial activation, neuroinflammation

## Abstract

**Introduction:**

Alzheimer's disease (AD) is the most common neurodegenerative disorder that causes dementia and affects millions of people worldwide. Although it has devastating outcomes for patients and tremendous economic costs to society, there is currently no effective treatment available.

**Aims:**

The high‐fat, low‐carbohydrate ketogenic diet (KD) is an established treatment for refractory epilepsy with a proven efficacy. Although the considerable interest has emerged in recent years for applying KD in AD patients, only few interventional studies in animals and humans have addressed the effects of KD on cognitive impairments, and the results were inconclusive. The aim of this study was to explore the impact of KD on cognitive functions and AD pathology in 5XFAD mice—a validated animal model of AD.

**Results:**

Four months of a ketogenic diet improved spatial learning, spatial memory and working memory in 5XFAD mice. The improvement in cognitive functions was associated with a restored number of neurons and synapses in both the hippocampus and the cortex. Ketogenic diet treatment also reduced amyloid plaque deposition and microglial activation, resulting in reduced neuroinflammation. The positive effect of ketogenic diet on cognitive functions depended on the starting time and the duration of the diet. A shorter period (2 months) of ketogenic diet treatment had a weaker effect. Ketogenic diet initiated at late stage of AD (9 months of age) displayed no effect on cognitive improvement.

**Conclusions:**

These findings indicate positive effects of ketogenic diet on both cognitive function and histopathology in Alzheimer's disease, which could be due to reduced microglial activation and neuroinflammation. Our findings provide new insights and therapeutic interventions for the treatment of Alzheimer's disease.

## Graphical abstract

Ketogenic diet improved cognitive functions, decreased amyloid plaque deposition and microglial activation, restored synaptic and neuronal density, and reduced neuroinflammation.

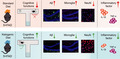

## 1 INTRODUCTION

Alzheimer's disease (AD) is the most common form of neurodegenerative dementia that is characterized by memory loss and impaired cognitive functions.^1^ There are 47 million people living with Alzheimer's dementia worldwide (https://www.alz.org/). AD typically occurred ~1 in 9 people older than 65 and ~1 in 3 people older than 85.^1^, ^2^ As the average life expectancy increases, the number of AD cases is expected to reach 76 million by 2030. Despite its devastating consequences and tremendous economic cost, there is no effective treatment thus far. Thus, AD becomes one of the most severe global public health issues.^1^

The key neuropathology of AD includes gross atrophy of the brain, deposition of amyloid plaques, neurofibrillary tangles, and extensive loss of neurons and synapses.^1^, ^3^ It is believed that the accumulation of Aβ is the primary factor that initiates multiple neurotoxic events and drives AD pathogenesis.^4^, ^5^, ^6^ Around Aβ plaques, gliosis is characterized by the proliferation and activation of astrocytes and microglia that cause inflammatory responses and contribute to neuronal and synapse loss. The loss of neurons and synapses eventually causes memory loss and cognitive impairment. Thus, many efforts have been made to clear Aβ in the brain.^4^, ^5^ However, many clinical trials targeting Aβ have yielded disappointing results.^7^ Recently, a therapy aiming at removing amyloid plaques by an Aβ‐directed antibody, aducanumab, has received accelerated approval as a treatment for Alzheimer's disease by the U.S. Food and Drug Administration (FDA).^8^ However, aducanumab is not a perfect cure, and although it can effectively clear Aβ, evidence that it can restore lost memories or cognitive function in AD patients remains to be collected.^8^, ^9^

The ketogenic diet (KD) is a high‐fat, low‐carbohydrate diet that promotes the genesis of ketone bodies (mainly acetoacetate and β‐hydroxybutyrate) through fat metabolism in the liver.^10^ KD has been clinically demonstrated to be effective in treating refractive epilepsy.^11^ It has been proposed that KD alter the composition of the gut microbiota^12^, ^13^ and increase GABA levels which might mediate the anti‐seizure effect.^14^ KD has also been reported to reduce neuroinflammation,^15^ which has been proposed to be beneficial to other neurological diseases. Although there are considerable interests in the impact of KD on AD progression, few studies have addressed whether KD could improve cognitive functions and the underlying mechanism.^16^

In this study, we subjected 5XFAD mice to different periods of a ketogenic diet that was initiated at different stages of AD. We found that a 4‐month KD initiated at 7 months of age dramatically improved the cognitive ability of AD model mice. The improvement in cognitive ability was associated with a restored number of synapses and neurons, and a reduction in Aβ deposition and microgliosis. Inflammation in the brain was also decreased by KD consumption. These results suggest that KD improves cognitive function, possibly by reducing multiple neuropathologies associated with AD.

## 2 MATERIALS AND METHODS

### 2.1 Animals

5XFAD mice were obtained from the Jackson Laboratory. 5XFAD transgenic mice express 5 human familial Alzheimer's disease (FAD) mutants in amyloid‐beta precursor protein (APP) and presenilin 1 (PS1) driven by the Thy1 promoter.^17^ Experimental 5XFAD mice were obtained by crossing heterozygous transgenic mice with C57BL/6 wild‐type breeders. Wild‐type littermates were used as controls and randomly allotted to each experiment. Male mice were used in all experiments. All mice were housed in a standard animal facility with a 12 h alternating light/dark cycle. All experiments in this study were approved by the Animal Care and Use Committees at the Shenzhen Institute of Advanced Technology (SIAT), Chinese Academy of Sciences (CAS).

### 2.2 Diets

Ketogenic diet formula (Patent NO.201210123235.0) was manufactured and supplied by Jintong Special Medical Food Co., Ltd (Guangzhou, China). It contained 76% fat, 16% protein, 3% carbohydrate, and 5% dietary fiber in kcal. The control diet was a standard laboratory chow diet from Beijing Keao Xieli Feed Co., Ltd (Beijing, China). It contained 12% fat, 23% protein, and 65% carbohydrate in kcal. Both the ketogenic diet and standard diet contained multiple vitamins and minerals. For the animals that were fed with a standard diet, the standard diet was available ad libitum. For the animals fed with a ketogenic diet, the ketogenic diet was provided at an amount that matched the calories to that of the standard diet consumed by wild‐type mice each day.

### 2.3 Barns maze

The protocol for the Barns maze was performed as previously described.^18^, ^19^ Briefly, the apparatus (Chengdu Techman Instrument Co., Ltd., ST‐120) was a revolvable white acrylic disc (0.75 m in diameter) 0.58 m elevated from the floor with 18 holes (5 cm diameter) equally spaced along the perimeter of the circle and positioned in a brightly light (600 lux). One hole was selected as the escape target hole, and a dark escape chamber was placed under the target hole consistent for each trial. Mice were allowed to freely explore on the platform for 5 min without the escape chamber, and habituated to the escape chamber for 2 min on two consecutive days. In the training phase, a session of 2 trials was performed by each mouse. Each mouse was covered under a nontransparent cylinder placed in the center of the maze. After 15 s, the cylinder was gently removed to allow mouse to explore the maze for 180 s until the target hole was found. If mice did not find the target hole, the latency was considered to be 180s. The maze and the escape chamber were wiped with 70% ethanol. The mouse was identified to find the target chamber when the back of the mice crossed the target hole. The mouse was considered to have entered the target chamber if the entire body was on the platform. The primary latency that mice took to find the target hole was documented for each trial. Two trials per day were performed for eight days. For the probe test, memory retention was assessed 5 days after the last training trial. The duration of the probe trial was 90 s. If the animal did not find the target hole within 90 s, the latency was considered as 90 s. The position of the target hole was the same as that in the training period. The primary latency to find the target hole and time in the target quadrant was analyzed by Anymaze software.

### 2.4 T Maze

T maze protocol was performed as previously described.^20^ Briefly, before performing the task, the diet of mice was restricted daily to hold 80% to 85% of their primary body weight throughout the task. The T maze apparatus was placed in a quiet room, and light intensity was held at a constant level (100 lux). During the training, a forced choice was followed by a free choice for each trial. In the forced‐choice phase, a food pellet was placed in either the left or right choice arm. The door to the choice arm with food pellet was opened, while the other choice arm was closed. The mouse was allowed to consume the pellet. When the pellet was consumed, the mouse was returned to the start arm. After the forced choice was completed, the free choice was initialized. During the free choice, all doors were open; the mouse was allowed to freely enter one of two arms. If the mouse entered the same arm as in the forced‐choice phase, the trial was considered to result in an “error,” and the pellet was removed. If the mouse entered the opposite arm as in the forced‐choice phase, the trial was considered to have a “correct” result, and the mouse was rewarded with a food pellet. Following each session, the apparatus was cleaned with 70% ethanol. Six consecutive trials were performed in a session per day. The percentage of correct responses was calculated in each session.

### 2.5 Open field test

Motor activity and anxiety were studied in the open field test. Briefly, mice were placed in a white Plexiglas box (40 × 40 × 40 cm) in the dark and were allowed to freely explore the arena. The mouse activity was monitored under an infrared camera for 5 min. The distance and time spent in the center zone and total distance were measured by Anymaze software. The box was cleaned with 70% ethanol thoroughly after each trial.

### 2.6 Immunofluorescence staining

Immunofluorescence staining was performed as previously described.^21^, ^22^ Mice were perfused transcardially with PBS and then 4% paraformaldehyde (PFA) in 0.1 M PBS sequentially. The brains were extracted and postfixed in 4% PFA overnight at 4°C, followed by dehydration in 15% and 30% sucrose until immersed at the bottom of the tube. Brains were embedded in O.C.T. Compound and sectioned into 40 μm thick slices collected in PBS using a 24‐well plate using a cryostat (Leica). For staining, brain sections were washed with PBS three times followed by blocking with 10% normal goat serum containing 0.3% Triton X‐100 for 2 h at room temperature in PBS. Subsequently, sections were incubated in primary antibody diluted in blocking solution (5% normal goat serum containing 0.3% Triton X‐100 in PBS) at 4°C for 24–48 h. The sections were washed three times with PBS and then incubated in secondary antibodies diluted in blocking solution (5% normal goat serum containing 0.3% Triton X‐100 in PBS) for 2 h at room temperature. Cell nuclei were stained with 6‐diamidino‐2‐phenylindole (DAPI). Afterward, sections were washed with PBS three times and mounted on slides. Fluorescent images were captured using a confocal microscope (Zeiss LSM 800) or slide scanner (Olympus, VS120). The primary antibodies used were as follows: mouse anti‐Aβ42 (1:500, Covance, #SIG‐39320, clone 12F4), rabbit anti‐Iba‐1 (1:500, Wako, # 019–19741), rabbit anti‐synaptophysin (1:500, Abcam, ab14692), mouse anti‐PSD95 (1:500, Origene, 75‐028), and mouse anti‐NeuN (1:500, Millipore, MAB377). The secondary antibodies used were Alexa Fluor 488 goat anti‐mouse, Alexa Fluor Cy5 goat anti‐rabbit, (1:500, Invitrogen), and Alexa Fluor Cy3 donkey anti‐mouse.

### 2.7 Enzyme‐linked immunosorbent assay (ELISA)

Mouse brain tissues were obtained after behavior testing. Briefly, mice were deeply anesthetized with pentobarbital, and the brains were collected on ice. Brain tissue was homogenized in RIPA lysis buffer containing additional protease inhibitor cocktail (Sigma) and 1 mM PMSF prior to centrifugation for 10 min at 13800 *g* (4°C). The supernatant was collected, and the total protein concentration evenly quantified to 2.5 mg/ml was measured using the Enhanced BCA Protein Assay kit (Beyotine Biotechnology). The concentrations of cytokines (IL‐1β and TNF‐α) were detected in duplicate with an ELISA kit (Neobioscience) according to the product protocol. All samples were stored at −80°C before the ELISA experiment. The protein concentration was determined with a microreader (BIOTEK Elx800).

### 2.8 Golgi staining

Golgi staining was performed as the manufacturer's instruction (FD, Neurotechnologies). Mice were deeply anesthetized with pentobarbital, and the brains were collected on ice. The brains were immersed in Golgi Solution A and B mixture for 2 weeks at room temperature and then replaced with Golgi Solution C at 4°C for 3 days. Coronal sections were cut at 150 μm with cryostat (Thermo Fischer Scientific). Brain slices were attached on gelatin‐coated slides and keeping being dry at room temperature. Brain slices were treated with Golgi Solution D/E. Subsequently, brain slices were dehydrated with 50%, 75%, 95%, and 100% alcohol prior to coverslipping. Images were obtained using a 100× oil immersion objective (Olympus BX53F2 upright fluorescence microscope). The spines in secondary dendritic branches (24 neurons in LEC from three mice) were calculated in Image J software. The density of spines was calculated by dividing the total number of spines to the length of dendritic branch (20 μm).

### 2.9 Statistical analysis

Data are expressed as the mean ±standard error of the mean (SEM). Statistical analysis was performed using GraphPad Prism software (version 8.0). The Shapiro‐Wilk test was used to test the normality. If the data were normally distributed, one‐way analysis of variance (ANOVA) or two‐way ANOVA was used to compared the differences between three independent samples, and the least significant difference test (LSD) was applied for post hoc multiple comparisons. Otherwise, nonparametric Kruskal‐Wallis tests were performed and followed by Dunn's post hoc tests. *p* Values <0.05 were considered statistically significant.

## 3 RESULTS

### 3.1 Ketogenic diet ameliorates cognitive impairment in 5XFAD mice

**FIGURE 1:**
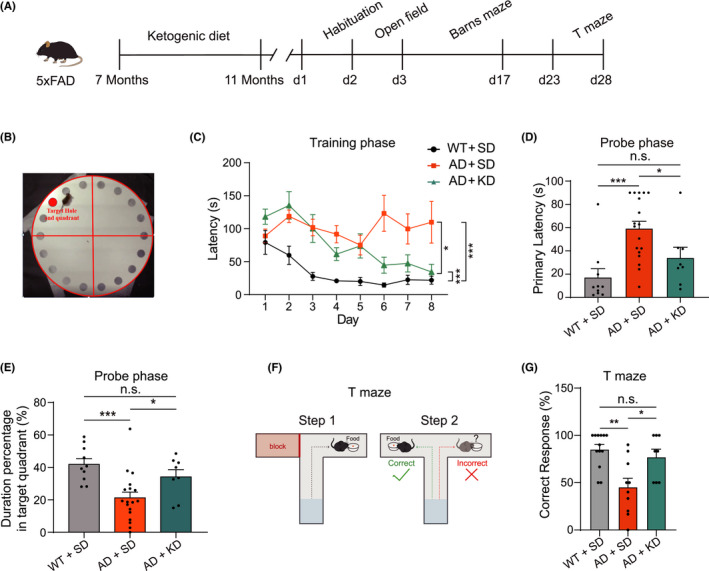
Ketogenic diet ameliorates cognitive impairment in 5XFAD mice. (A) Schematic showing the experiment procedure. The 5XFAD mice were fed with a ketogenic diet at the age of 7 months, and cognitive behavior tests were performed at the age of 11 months. (B) Schematic showing the Barns maze for the spatial learning and memory tests. (C) The latency to find the escape hole during the training phase of the Barns maze for WT + SD group (black, *n* = 10), AD + SD group (orange, *n* = 18), and AD + KD group (cyan, *n* = 8). Two‐way ANOVA test: *p* < 0.05; Least significant difference test: *p < 0.05 and ***p < 0.001. (D) The average latency to find the escape hole during the probe test of the Barns maze for WT + SD group (black, *n* = 8), AD + SD group (orange, *n* = 18), and AD + KD group (cyan, *n* = 8). One‐way ANOVA test: *p* < 0.001; Least significant difference test: *p < 0.05, ***p < 0.001, n.s., no significant difference. (E) The time spent in the target quadrant during the probe test of the Barns maze for WT + SD group (black, *n* = 8), AD + SD group (orange, *n* = 18), and AD + KD group (cyan, *n* = 8). One‐way ANOVA test: *p* < 0.01; Least significant difference test: *p < 0.05, ***p < 0.001, n.s., no significant difference. (F) Schematic showing the T maze for the working memory test. (G) The percentage of correct trials in the T maze for WT + SD group (black, *n* = 13), AD + SD group (orange, *n* = 10), and AD + KD group (cyan, *n* = 8). Kruskal‐Wallis test: *p* < 0.01; Dunn's post hoc test: *p < 0.05, **p < 0.01, n.s., no significant difference

The overall experimental procedure is illustrated in Figure 1A. 5XFAD mice started a ketogenic diet (KD) at the age of 7 months—when they already exhibited severe AD histopathology and cognitive decline.^23^ The control 5XFAD mice and wild‐type littermates were fed with a standard laboratory chow diet (standard diet, SD). Cognitive function was tested 4 months after KD, at the age of 11 months for all groups of mice (Figure 1A). We firstly assessed hippocampus‐dependent spatial learning and memory with the Barns maze,^24^, ^25^ a dry‐land version of the Morris water maze (Figure 1B). During the training phase, wild‐type mice fed with SD showed a progressive decrease in the latency to find the escape hole, indicating significant learning over 8 days of training (Figure 1C, black curve). The 5XFAD mice fed with SD had a much longer latency and did not demonstrate this improvement, suggesting a severe deficit in the spatial learning task (Figure 1C, orange curve). In contrary, 5XFAD mice fed with KD showed significant improvement in spatial learning (Figure 1C, cyan curve). A two‐way mixed analysis of variance (ANOVA) (Group X Training Day) on latency revealed a main effect of training day (time: *F*(7,225) = 5.9, *p* < 0.001; group: *F*(2,225) = 46.2, *p* < 0.001), and interaction (*F*(14,225) = 2.2, *p* < 0.001). On the final day (8th day) of training, 5XFAD mice fed with KD had a much shorter latency (34.4 ± 11.6 s) than those fed with SD (109.9 ± 31.5 s) and was not different from wild‐type mice fed with SD (22.0 ± 6.0 s) (Figure 1C).

A probe test was performed to assess memory recall for the escape hole location 5 days after Barns maze training. During the probe phase, 5XFAD mice fed with SD showed much longer primary latency (59.2 ± 6.3 s) compared with the wild‐type mice (17.2 ± 7.5 s), and this impaired memory retention was significantly corrected by KD (34 ± 9.2 s) (Figure 1D). The percentage of time spent in the target quadrant during the 90 s probe trial was also significantly increased in 5XFAD mice fed with KD (34.2% ± 4.4%) compared with those fed with SD (21.2% ± 3.5%) (Figure 1E). These results indicate that 5XFAD mice had impaired spatial learning and memory, and this impairment was alleviated by a 4‐month ketogenic diet.

We next examined working memory in the T maze task (Figure 1F), which takes advantage of rodents’ spontaneous alternation nature.^26^, ^27^, ^28^ The percentage of correct trials was markedly decreased in the 5XFAD mice (45.3% ± 9.3%) compared with the wild‐type mice (85.1% ± 5.2%), and this deficit was markedly rescued by KD (77.1% ± 8.3%) (Figure 1G). These results indicate that impaired spatial working memory in 5XFAD mice was restored by 4 months of KD treatment.

**FIGURE 2:**
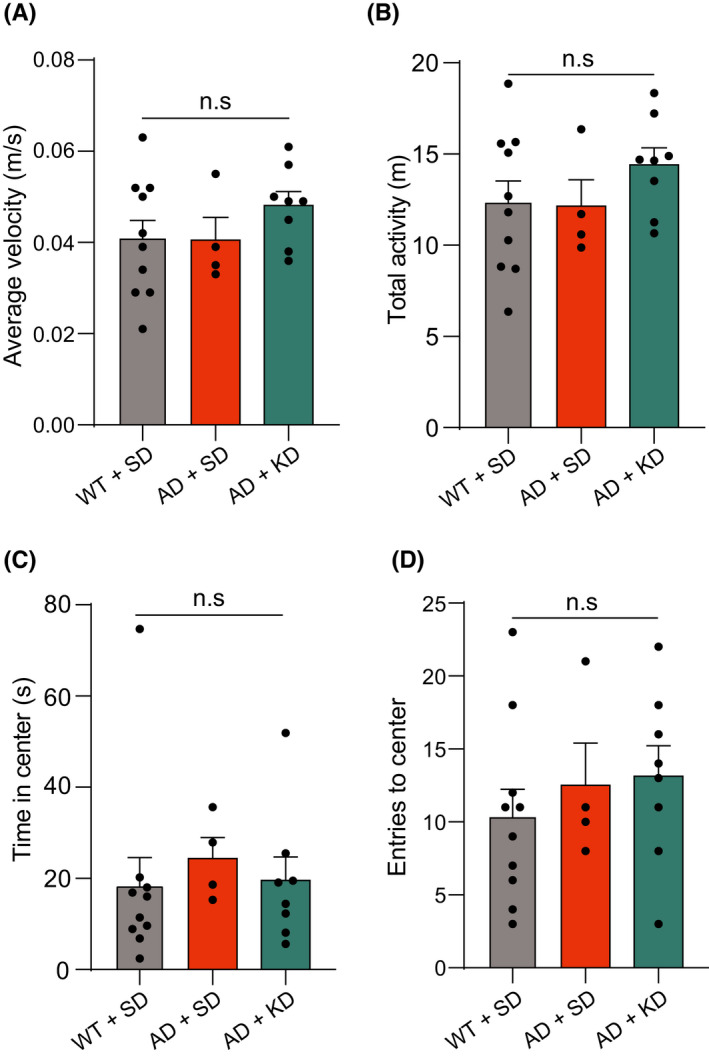
Impact of ketogenic diet on locomotor activity and anxiety level in 5XFAD mice. (A) The average velocity in the open field test for WT + SD group (black, *n* = 10), AD + SD group (orange, *n* = 4), and AD + KD group (cyan, *n* = 8). One‐way ANOVA test: *p* > 0.05; Least significant difference test: n.s., no significant difference. (B) The total activity in the open field test for WT + SD group (black, *n* = 10), AD + SD group (orange, *n* = 4), and AD + KD group (cyan, *n* = 8). One‐way ANOVA test: *p* > 0.05; Least significant difference test: n.s., no significant difference. (C) The time spent in the center in the open field test for WT + SD group (black, *n* = 10), AD + SD group (orange, *n* = 4), and AD + KD group (cyan, *n* = 8). One‐way ANOVA test: *p* > 0.05; Least significant difference test: n.s., no significant difference. (D) The entries to the center in the open field test for WT + SD group (black, *n* = 10), AD + SD group (orange, *n* = 4), and AD + KD group (cyan, *n* = 8). Kruskal‐Wallis test: *p* > 0.05; Dunn's post hoc test: n.s., no significant difference

We also tested locomotor activity and anxiety levels in the open field task. The average velocity and total locomotor activity did not show differences between wild‐type and 5XFAD mice fed with SD, or 5XFAD mice fed with KD (Figure 2A,B). The time in the center and entries to the center also did not differ between wild‐type and 5XFAD mice fed with SD, and 5XFAD mice fed with KD (Figure 2C,D). These results suggest that the improvement in cognitive functions could not be due to changes in locomotor activity or anxiety levels.

### 3.2 Ketogenic diet prevents synaptic and neuronal loss in 5XFAD mice

**FIGURE 3:**
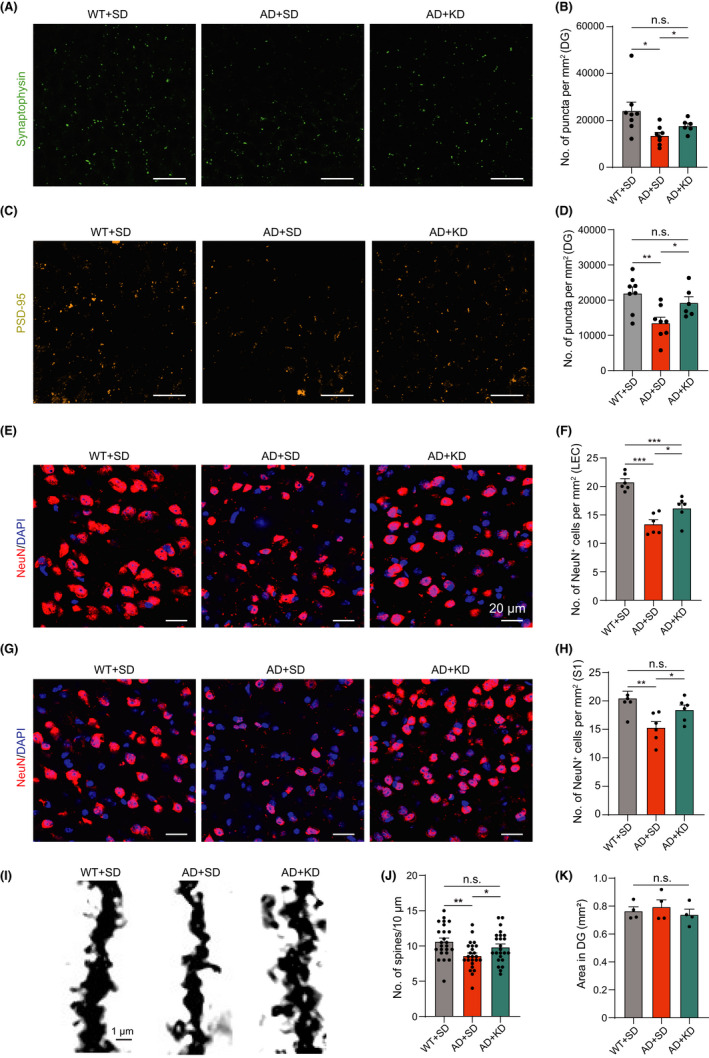
Ketogenic diet prevents synaptic and neuronal loss in 5XFAD mice. (A) Representative confocal images of synaptophysin in the DG area of the hippocampus from mouse in WT + SD group (left), AD + SD group (middle), and AD + KD group (right). Scale bar, 20 μm. (B) Average number of synaptophysin‐positive puncta per mm^2^ in DG for WT + SD group (black, *n* = 8), AD + SD (orange, *n* = 8), and AD + KD group (cyan, *n* = 6). One‐way ANOVA test: *p* < 0.05; Unpaired t with Welch's correction: *p < 0.05, n.s., no significant difference. (C) Representative confocal images for PSD‐95 from DG area of hippocampus from mouse in WT + SD group (left), AD + SD group (middle), and AD + KD group (right). Scale bar, 20 μm. (D) Average number of PSD95‐positive puncta per mm^2^ in the DG for WT + SD group (black, *n* = 8), AD + SD group (orange, *n* = 8), and AD + KD group (cyan, *n* = 6). One‐way ANOVA test: *p* < 0.01; Least significant difference test: **p < 0.01, *p < 0.05, n.s., no significant difference. (E) Representative confocal images of NeuN (red) in the lateral entorhinal cortex (LEC) from mouse in WT + SD group (left), AD + SD group (middle), and AD + KD group (right). Scale bar, 20 μm. (F) Average number of NeuN^+^ cells per mm^2^ in LEC for WT + SD group (black, *n* = 6), AD + SD group (orange, *n* = 6), and AD + KD group (cyan, *n* = 6). One‐way ANOVA test: *p* < 0.001; Least significant difference test: ***p < 0.001, *p < 0.05. (G) Representative confocal images of NeuN (red) in the somatosensory cortex from mouse in WT + SD group (left), AD + KD group (middle), and AD + KD group (right). Scale bar, 20 μm. (H) Average number of NeuN^+^ cells per mm^2^ in somatosensory cortex for WT + SD (black, *n* = 6), AD + SD group (orange, *n* = 6), and AD + KD group (cyan, *n* = 6). One‐way ANOVA test: *p* < 0.05; Unpaired t with Welch's correction: **p < 0.01, *p < 0.05, n.s., no significant difference. (I) Representative images of dendritic spines in secondary branch of hippocampal DG neuron from mouse in WT + SD group (left), AD + SD group (middle), and AD + KD group (right). Scale bar, 1 μm. (J) Average spine density of hippocampal DG for WT + SD group (black, *n* = 24), AD + SD group (orange, *n* = 24), and AD + KD group (cyan, *n* = 24). One‐way ANOVA test: *p* < 0.01; Unpaired *t* with Welch's correction: **p < 0.01, *p < 0.05, n.s., no significant difference. (K) Area of hippocampal DG for WT + SD group (black, *n* = 4), AD + SD group (orange, *n* = 4), and AD + KD group (cyan, *n* = 4). One‐way ANOVA test: *p* > 0.05; Least significant difference test: n.s., no significant difference

Synapse loss is highly correlated with cognitive declines in Alzheimer's disease and is believed to be the pathological basis of cognitive impairment.^29^, ^30^, ^31^ In 5XFAD mice, significant synaptic loss was detected at 6 months of age.^32^, ^33^ We firstly assessed the effect of KD on synapses by immunohistochemical staining for the presynaptic vesicle protein synaptophysin.^34^ The 5XFAD mice showed a dramatic decrease in this presynaptic marker in both the hippocampus (Figure 3A,B) and neocortex (Figure S1A,B) compared with wild‐type mice, confirming massive synaptic loss during AD progression. KD treatment notably reversed the decrease in synaptophysin in 5XFAD mice (Figures 3A,B and S1A,B). We further confirmed this finding by immunostaining for the postsynaptic marker PSD‐95, and obtained similar results (Figures 3C,D and S1C,D). These results indicate ketogenic diet inhibits the loss of synapse in 5XFAD mice.

Neuronal loss is another prominent feature of AD pathology that contributes to cognitive impairment.^1^ In 5XFAD mice, noticeable neuronal loss was observed as early as 9 months,^17^ and approximately 40% of layer V pyramidal neurons were lost at the age of 12 months.^35^ We next examined neuronal density by performing immunostaining for the neuronal marker NeuN. The number of NeuN^+^ cells per mm^2^ was significantly reduced in both the lateral entorhinal cortex (LEC, 13.4 ± 0.7), and somatosensory cortex (S1, 15.3 ± 1.1) in 5XFAD mice compared with wild‐type littermates (LEC: 20.7 ± 0.6; S1: 20.5 ± 1.2), confirming overall neuronal loss during AD progression. The number of NeuN^+^ cells was largely restored by KD (LEC: 16.2 ± 0.9; S1: 18.5 ± 0.8) (Figure 3E–H). The findings indicate that KD prevents neuronal loss in 5XFAD mice.

Since neuronal loss was also observed, it raised the possibility that whether synaptic loss was purely due to the neuronal loss or was also contributed by synaptic loss in each survival neuron. We then performed Golgi staining^36^ to examine spine density of individual neuron. The spine density of secondary branch of individual neuron was significantly decreased in 5XFAD mice (8.6 ± 0.4 per 10 μm) compared with wild‐type littermates (10.6 ± 0.5 per 10 μm). However, the spine density was largely restored by KD (9.8 ± 0.5 per 10 μm) (Figure 3I,J). Moreover, there is no difference in the size of hippocampal dentate gyrus (DG) area among three groups (Figure 3K).

### 3.3 Ketogenic diet reduces Aβ deposition in 5XFAD mice

**FIGURE 4:**
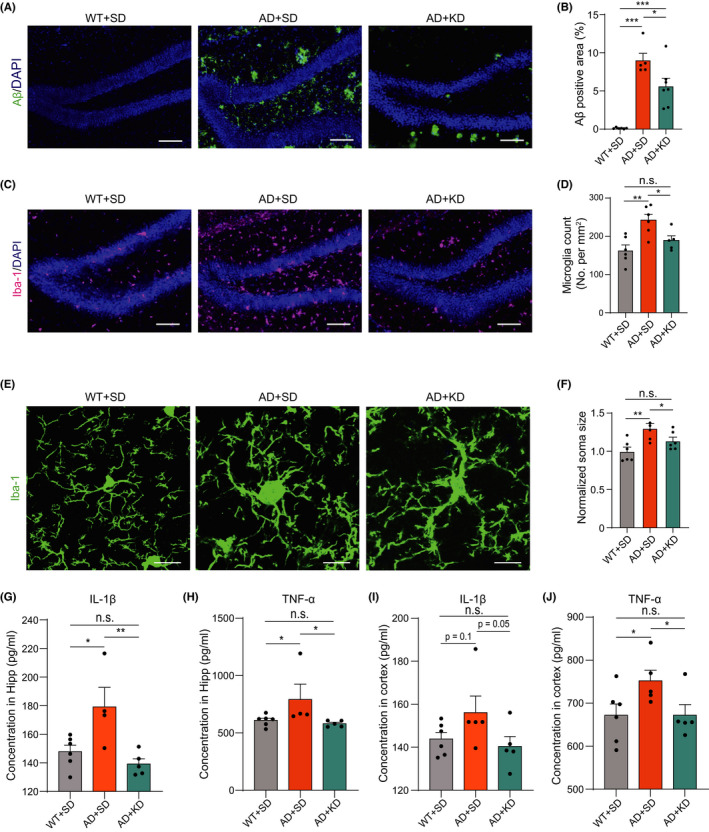
Ketogenic diet reduced Aβ deposition and microgliosis in 5XFAD mice. (A) Representative images of Aβ42 (green) from in the DG area of the hippocampus from mouse in WT + SD group (left), AD + SD (middle), and AD + KD (right). Scale bar, 100 μm. (B) Average area of Aβ plaque per mm^2^ in the DG for WT + SD group (black, *n* = 6), AD + SD group (orange, *n* = 5), and AD + KD group (cyan, *n* = 7). One‐way ANOVA test: *p* < 0.001; Least significant difference test: *p < 0.05, ***p < 0.001. (C) Representative images of microglia labeled by Iba‐1 immunostaining (magenta) in the DG area of the hippocampus from mouse in WT + SD group (left), AD + SD group (middle), and AD + KD group (right). Scale bar, 100 μm. (C) Average number of Iba‐1^+^ cells per mm^2^ in the DG for WT + SD group (black, *n* = 6), AD + SD group (orange, *n* = 6), and AD + KD group (cyan, *n* = 5). One‐way ANOVA test: *p* < 0.001; Least significant difference test: **p < 0.01, *p < 0.05, n.s., no significant difference. (D) Representative images of microglia labeled by Iba‐1 immunostaining (green) from mouse in WT + SD group (left), AD + SD group (middle), and AD + KD group (right). Scale bar, 10 μm. (E) Quantification of soma size of Iba‐1^+^ cells from WT + SD group (black, *n* = 6), AD + SD group (orange, *n* = 6), and AD + KD group (cyan, *n* = 6). One‐way ANOVA test: *p* < 0.01; Least significant difference test: **p < 0.01, *p < 0.05, n.s., no significant difference. (G–H) Concentrations of IL‐1β (G) and TNF‐α (H) in hippocampal brain tissue from WT + SD group (black, *n* = 6), AD + SD group (orange, *n* = 5), and AD + KD group (cyan, *n* = 5). IL‐1β: one‐way ANOVA test: *p* < 0.01; Least significant difference test: **p < 0.01, *p < 0.05, n.s., no significant difference; TNF‐α: Kruskal‐Wallis test: *p* < 0.05; Dunn's post hoc test: *p < 0.05, n.s., no significant difference. (I–J) Concentrations of IL‐1β (I) and TNF‐α (J) in cortical brain tissue from WT + SD group (black, *n* = 6), AD + SD group (orange, *n* = 5), and AD + KD group (cyan, *n* = 5). IL‐1β: one‐way ANOVA test: *p* > 0.05; Least significant difference test: n.s., no significant difference; TNF‐α: Kruskal‐Wallis test: *p* < 0.05; Dunn's post hoc test: *p < 0.05, n.s., no significant difference

The accumulation of Aβ is thought to initiate multiple neurotoxic events, including synaptic and neuronal loss that lead to neurodegeneration.^13^, ^14^ Therefore, we examined whether KD affected Aβ plaques in 5XFAD mice by immunohistochemical staining of Aβ42. The 5XFAD mice showed significant Aβ deposition increase at 11 months of age. Strikingly, Aβ deposition in the hippocampus was significantly decreased in 5XFAD mice fed with KD compared with those fed with SD (Figure 4A,B). These observations suggest that KD prevents Aβ deposition in 5XFAD mice.

### 3.4 Ketogenic diet decreases microgliosis and neuroinflammation in 5XFAD mice

The accumulation of Aβ induces neuroinflammation and activates microglia.^37^, ^38^ The activation of microglia has been proposed to mediate synaptic loss through complement‐dependent synapse engulfment during AD progression.^39^, ^40^ We then assessed microglial functions by immunstaining for Ionized calcium‐binding adaptor molecule 1 (Iba‐1), a molecular marker of microglia.^41^, ^42^ The number of Iba‐1^+^ cells per mm^2^ was significantly increased in 5XFAD mice (242.3 ± 15.3) compared with wild‐type littermates (163.2 ± 14.3), consistent with the microgliosis pathology of AD. In contrast, KD treatment significantly reduced Iba‐1^+^ cells (189.1 ± 20.0) (Figure 4C,D).

Activated microglia undergoes morphological changes and engulfs synapses,^43^ which is one of the mechanisms by which microglia mediate synapse loss during AD progression. We thus examined the morphology of microglia. The microglial cell body became larger (Figure 4E,F), and the primary process became shorter (Figure S2A) in 5XFAD mice compared with wild‐type mice, indicating a switch toward a phagocytic state of microglia.^44^, ^45^ Strikingly, KD significantly decreased the microglial cell body diameter and increased the primary process length. These results indicate that KD attenuates microglial activation and alleviates microgliosis.

Neuroinflammation contributes to AD pathogenesis.^46^ To further examine whether KD reduces neuroinflammation, we examined the levels of the pro‐inflammatory cytokines interleukin‐1β (IL‐1β) and tumor necrosis factor‐α (TNF‐α) in the hippocampal and neocortical tissue by ELISA. IL‐1β and TNF‐α concentrations in both the hippocampus and neocortex were increased in 5XFAD mice compared with wild‐type mice, suggesting excessive neuroinflammation in subjects with AD. However, this increase in IL‐1β and TNF‐α was reversed by KD treatment (Figure 4G–J). These results indicate that KD reduces the inflammation response in the brain.

### 3.5 The improvement effect of ketogenic diet on cognition depends on the time and duration of intervention

**FIGURE 5:**
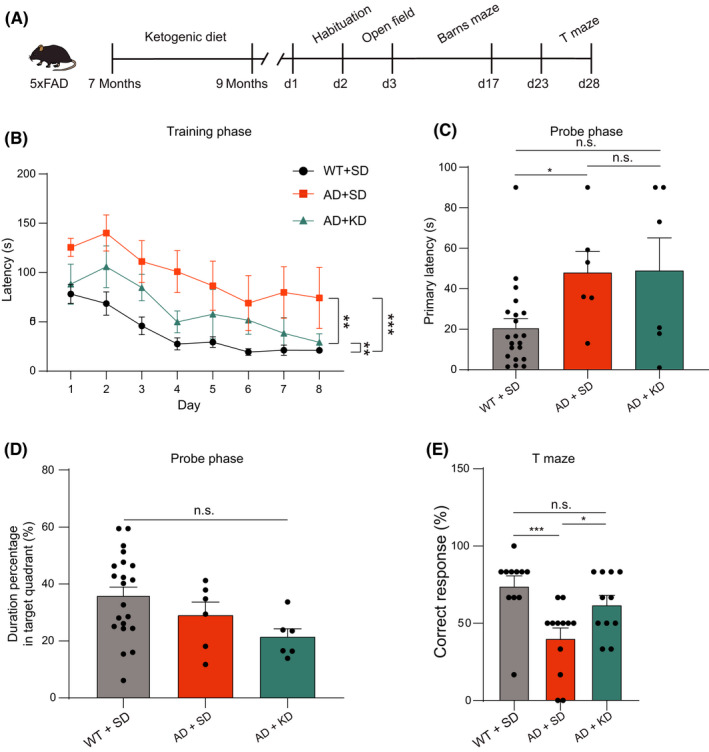
Impact of 2 months (7–9 months of age) of ketogenic diet on cognitive function in 5XFAD mice. (A) Schematic showing the experiment procedure. The 5XFAD mice were fed with a ketogenic diet at the age of 7 months for 2 months. (B) The latency to find the escape hole during the training phase of the Barns maze for WT + SD group (black, *n* = 21), AD + SD group (orange, *n* = 6), and AD + KD group (cyan, *n* = 6). Two‐way ANOVA test: *p* < 0.001; Least significant difference test: ***p < 0.001, **p < 0.01. (C) The average latency to find the escape hole during the probe test of the Barns maze for WT + SD group (black, *n* = 21), AD + SD group (orange, *n* = 6), and AD + KD group (cyan, *n* = 6). Kruskal‐Wallis test: *p* < 0.05; Dunn's post hoc test: *p < 0.05, n.s., no significant difference. (D) The time spent in the target quadrant during the probe test of the Barns maze for WT + SD group (black, *n* = 21), AD + SD group (orange, *n* = 6), and AD + KD group (cyan, *n* = 6). One‐way ANOVA test: *p* > 0.05; Least significant difference test: n.s., no significant difference. (E) The percentage of correct trials in the T maze for WT + SD group (black, *n* = 11), AD + SD group (orange, *n* = 12), and AD + KD group (cyan, *n* = 11). Kruskal‐Wallis test: *p* < 0.01; Dunn's post hoc test: ***p < 0.001, *p < 0.05, n.s., no significant difference

Our data, which showed that a 4‐month KD improved learning and memory in 5XFAD mice, led us to determine whether a shorter period of KD would also have a similar beneficial effect. We then subjected 5XFAD mice to KD for 2 months beginning at the age of 7 months (Figure 5A) and 9 months (Figure S3A). After 2 months of KD treatment, the spatial learning was only marginally improved during training phase of the Barns maze (Figures 5B and S3B). During the probe test, both the primary latency and time spent in the target quadrant did not differ in 5XFAD mice fed with KD vs. SD (Figures 5C,D and S3C,D). However, the percentage of correct trials in the T maze task was significantly increased in 5XFAD mice fed with KD (Figure 5E: 62.1% ± 6.0%; Figure S3E: 66.3% ± 7.0%) compared with those fed with SD (Figure 5E: 40.3% ± 6.6%; Figure S3E: 39.6% ± 10.9%). These results suggested that a 2‐month KD treatment improved working memory, but only marginally improved spatial learning and memory in 5XFAD mice.

**FIGURE 6:**
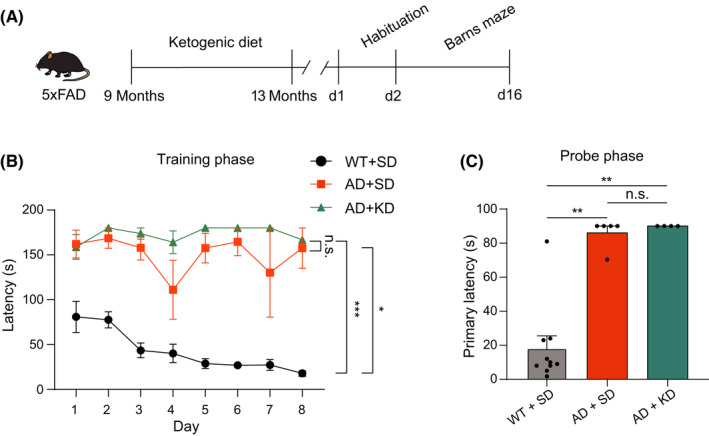
Impact of ketogenic diet started at the late stage on cognitive function in 5XFAD mice. (A) Schematic showing the experiment procedure. The 5XFAD mice were fed with ketogenic diet at the age of 9 months for 4 months. (B) The latency to find the escape hole during the training phase of the Barns maze for WT + SD group (black, *n* = 10), AD + SD group (orange, *n* = 5), and AD + KD group (cyan, *n* = 7). Two‐way ANOVA test: *p* < 0.05; Least significant difference test: *p < 0.05, ***p < 0.001, n.s., no significant difference. (C) The average latency to find the escape hole during the probe test of the Barns maze for WT + SD group (black, *n* = 10), AD + SD group (orange, *n* = 5), and AD + KD group (cyan, *n* = 4). Kruskal‐Wallis test: *p* < 0.001; Dunn's post hoc test: **p < 0.01, n.s., no significant difference

Finally, we determined whether starting KD at a late stage of AD progression could also exhibit the beneficial effects. Therefore, KD was initiated at the age of 9 months in 5XFAD mice and maintained for 4 months (Figure 6A). Spatial learning and memory were assessed by the Barns maze. However, the primary latency showed no difference between 5XFAD mice fed with KD and those fed with SD, during both the training phase and probe phase (Figure 6B,C). Thus, KD starting at a very late stage of AD does not improve cognitive functions in 5XFAD mice.

## 4 DISCUSSION

Alzheimer's disease is the leading cause of dementia in elderly people.^1^ Patients with AD experience progressive loss of memory and cognitive function, eventually leading to the disruption of daily life.^47^ Currently available treatments for AD only slightly delay the progression of the disease and do not affect the main neuropathological features of the disease.^48^ Numerous efforts have been made to develop drugs against AD pathogenesis, including Aβ, but the results were disappointing.^7^

Our results in the present study demonstrated that 4 months of ketogenic diet improved cognitive functions in 5XFAD mice. In addition to improvements in cognitive functions, synapse loss and neuronal loss were reversed. KD also attenuated Aβ deposition, decreased microgliosis, and reduced neuroinflammation. These results suggest that KD is able to alleviate AD pathology and improve learning and memory, probably by protecting neurons and synapses through reducing neuroinflammation and neurotoxic Aβ accumulation. Considering its proven efficacy and safety in treating refractive epilepsy,^4^ KD represents a very promising therapeutic intervention for managing Alzheimer's disease.

The exact link between KD and AD remains obscure. The ketogenic diet is a high‐fat, low‐carbohydrate diet that is able to promote ketogenesis. When glucose is not readily available, the brain's metabolism switches toward ketone bodies, which are generated through ketosis that break down fat in the liver.^31^ There are several mechanisms that could potentially underlie the beneficial effect of KD on AD. First, abnormal glucose metabolism was observed in advance of the onset of cognitive decline in AD.^49^ PET imaging studies revealed that the utilization of glucose declines in AD brain but ketone body utilization does not.^50^ Thus, KD might improve the cognition by providing an additional fuel supply to the brain. Second, apolipoprotein E (Apo‐E) is one of the most detrimental AD genetic risk factor.^51^, ^52^ The Apo‐E ε4 allele is associated with lower efficiency in lipid transport and increases AD risk. Thus, KD might alleviate AD symptoms by promoting lipid metabolism. Third, it has been shown that KD could alter gut microbiota.^14^ The gut microbiota regulates host brain functions via the gut‐brain axis, which has been reported to play an important role in AD pathogenesis.^53^, ^54^, ^55^, ^56^ Thus, KD might ameliorate AD pathology by regulating gut microbiota and reducing neuroinflammation.^44^, ^57^ In the future, it would be very interesting to examine the precise underlying mechanism of KD on the cognitive functions.

In 5XFAD mice, amyloid plaque is detected at the age of 2 months, synapse loss and cognitive impairments are observed at 6 months, and extensive neuronal loss occurs at 1 year. Our results show that 4‐month KD initiated at 7 months dramatically improves cognitive functions and alleviates AD pathology. This suggests that KD intervention upon the diagnosis of AD could display strong therapeutic effects. However, KD initiated at later stage of 9 months of age exhibits no beneficial effect, suggesting earlier intervention achieves better beneficial effect. Shorter intervention shows some detectable improvement, although it is weaker than longer intervention. Conceivably, it is very important to determine a proper KD protocol in AD patients to achieve the best effect on cognition.

## CONFLICT OF INTEREST

None.

## AUTHOR CONTRIBUTIONS

Y.Z. conceived the study. Y.X., C.J., J.W., and P.L. performed experiments. Y.X., J.W., and P.L. analyzed data. Y.Z. and Y.X. wrote the manuscript. Y.X., X.D., Y.D.Z., and B.P. revised and final proof the manuscript. All authors reviewed the manuscript.

## Supporting information

Figure S1

Figure S2

Figure S3

## Data Availability

The data that support the findings of this study are available from the corresponding author upon reasonable request.
